# Potassium-Modulated Ni Catalysts for Enhanced Hydrogen Production from Textile Waste via Microwave Pyrolysis

**DOI:** 10.3390/molecules31142443

**Published:** 2026-07-12

**Authors:** Xiange Wu, Yuxing Huang, Bo Zhang, Junhao Chen, Jingran Xia, Rui Bai, Wuwan Xiong

**Affiliations:** College of Environmental and Chemical Engineering, Zhaoqing University, Zhaoqing 526061, China; xiangewu@163.com (X.W.); hyx20050316@163.com (Y.H.); 15626687118@163.com (J.C.); 13672346824@163.com (J.X.); 15089189590@163.com (R.B.)

**Keywords:** waste textiles, potassium promotion, Ni-based catalyst, microwave pyrolysis, hydrogen production

## Abstract

The conversion of waste textiles into valuable products is an effective route to mitigate low-value solid waste accumulation and recover energy. In this work, nickel and potassium were introduced into textile waste via an impregnation method, and their roles in microwave-assisted catalytic pyrolysis were investigated with a focus on hydrogen production. The results show that co-loading 1 wt.% Ni and 0.4 wt.% K significantly enhances gas formation, with a total gas yield of 67.47% and a hydrogen yield of 52.57 mmol/g. Hydrogen production was markedly improved compared with the untreated textiles, the physical mixing methods, and the conventional pyrolysis. Structural characterization by XRD and SEM mapping confirmed that K addition effectively suppressed the agglomeration of Ni species. FTIR analysis suggests that a synergistic catalytic effect between K and Ni promotes the conversion of macromolecular components into smaller gaseous products. The improved hydrogen production can be associated with the combined effect of enhanced Ni dispersion and promoted decomposition reactions. This work provides new insights into the design of alkali-promoted Ni catalysts for efficient hydrogen production from textile waste under microwave pyrolysis conditions.

## 1. Introduction

The rapid accumulation of textile waste has become an increasingly serious environmental issue due to its large generation and low recycling efficiency [1,2,3]. Physical recycling technology has limited applicability to blended textiles and low recovery efficiency, while chemical recycling technology suffers from high energy consumption and secondary pollution [4,5,6,7,8]. Converting textile waste into value-added products through thermochemical processes offers a promising route for sustainable waste management [9,10]. Pyrolysis has attracted significant attention because waste textiles contain abundant carbonaceous organic matter and cellulose and their conversion via thermochemical methods can efficiently extract hydrogen energy, offering both resource recycling and carbon emission reduction potential [9,11]. In particular, hydrogen-rich gas produced via pyrolysis is of great interest due to its high energy density and clean combustion characteristics. 

Conventional pyrolysis relies on external heat sources, which can lead to uneven heating and the formation of large amounts of tar by-products, thereby reducing hydrogen yield and purity [12,13,14,15]. Microwave-assisted pyrolysis has emerged as an advanced technique compared to conventional heating methods, owing to its rapid and volumetric heating, high energy efficiency, and selective interaction with materials [16,17,18,19]. These advantages make microwave pyrolysis especially suitable for enhancing gas production from solid wastes. Microwave pyrolysis can utilize the dielectric loss of the material itself to achieve simultaneous internal and external heating, with a heating rate 3–5 times faster than conventional methods, and it preferentially breaks C-H and C-C bonds [10,20]. However, the intrinsic limitations of textile waste, such as complex composition and low reactivity, often result in limited hydrogen yield. Catalysts are therefore widely introduced to promote cracking reactions and improve product distribution.

Catalytic pyrolysis using metal salts is an effective strategy for achieving high-value utilization of waste textiles. Ren et al. indicated that, in addition to the relevant instrument parameters of microwave pyrolysis, leveraging the characteristics of catalysts is very important in microwave pyrolysis [21]. Among various catalysts, Ni-based materials are considered highly effective for hydrogen production due to their strong ability to facilitate C-C and C-H bond cleavage [10,22,23]. Nevertheless, Ni catalysts are prone to particle agglomeration and sintering under high-temperature pyrolysis conditions, which leads to the loss of active sites and deterioration of catalytic performance. Feng et al. studied the multiscale interaction between the external transition metal Ni and self-contained potassium in the fast/slow pyrolysis of biomass [23]. The results show that K can indirectly enhance the interaction between Ni and biochar supports by enhancing the oxygen-containing functional groups of biochar supports. Therefore, the introduction of alkali metals has emerged as an effective strategy for regulating catalyst structure and enhancing catalytic activity. Nevertheless, the role of K in modifying the structure of Ni catalysts and its impact on hydrogen evolution during the microwave pyrolysis of textile waste has not been fully elucidated and deserves further investigation.

In this study, K-promoted Ni catalysts were developed to enhance hydrogen production from textile waste via microwave pyrolysis. The effect of K introduction on Ni particle size, and product distribution was systematically investigated. Combined with the data from the pyrolysis evaluation, the active center of the metal salt catalysis was deduced. In addition, the performance evaluation data of conventional pyrolysis and microwave pyrolysis on the treatment of waste textiles were compared. Structural characterization using X-ray diffraction, Raman, scanning electron microscopy (SEM), and Fourier transform infrared spectroscopy (FTIR) was conducted to elucidate the changes in catalyst properties and reaction behavior. The relationship between catalyst structure and hydrogen evolution was further discussed, providing insights into the mechanism of K-modified Ni catalysts in microwave-assisted pyrolysis systems. This work provides a simple and effective strategy for improving hydrogen production from solid waste via catalyst design.

## 2. Results and Discussion

**Figure 1:**
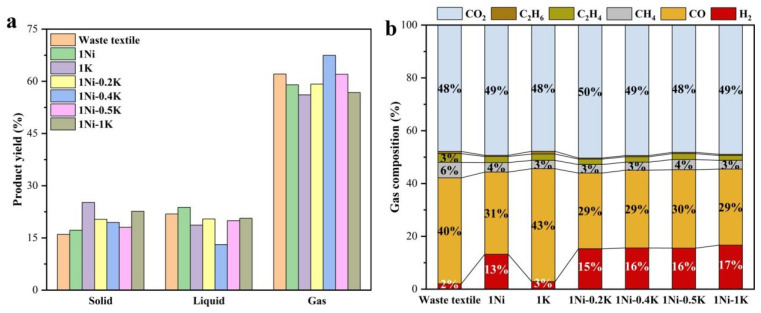
(**a**) Effect of metal salt species and content on the catalysis yield of three phase products of textile; (**b**) effect of metal salt species and content on the catalysis yield of gas phase products of textile.

To investigate the effect of K addition on the hydrogen production performance of Ni-containing samples during microwave pyrolysis, the product distributions under different Ni/K ratios were compared (Figure 1). From the three-phase product distribution in Figure 1a, it can be seen that the gas yield of pure waste textile was 62.09%, the liquid yield was 21.87%, and the solid yield was 16.04%. After adding 1 wt.% Ni, the gas yield of 1Ni sample decreased slightly to 59.00%, the liquid yield increased to 23.78%, and the solid yield increased to 17.21%. With 1 wt.% K, the gas, liquid, and solid yields of the 1K samples were 56.13%, 18.68%, and 25.19%, respectively. When Ni and K were co-loaded, the gas yield increased significantly, among which the 1Ni-0.4K sample achieved the highest gas yield (67.47%) and the lowest liquid yield (13.09%), with a solid yield of 19.44%. These results indicate that an appropriate amount of K addition helps promote the conversion to gaseous products and reduces the formation of liquid by-products. From the gas composition in Figure 1b, the H_2_ content of waste textile in the gas phase was only 2%. The 1K sample showed a negligible increase to just 3%, whereas the introduction of 1 wt.% Ni significantly raised the H_2_ content to 13%. When Ni and K were co-loaded, the H_2_ selectivity was further enhanced. This indicates that Ni is the key element promoting H_2_ generation and the introduction of K facilitated the conversion of liquid-phase compounds into gaseous products, leading to a higher gas yield and improved hydrogen production.

**Figure 2:**
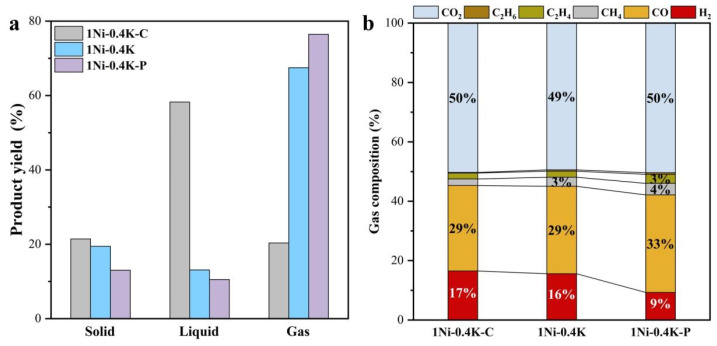
(**a**) Effect of impregnation and pyrolysis method on the catalysis yield of three phase products of textile; (**b**) effect of impregnation and pyrolysis method on the catalysis yield of gas phase products of textile.

**Figure 3:**
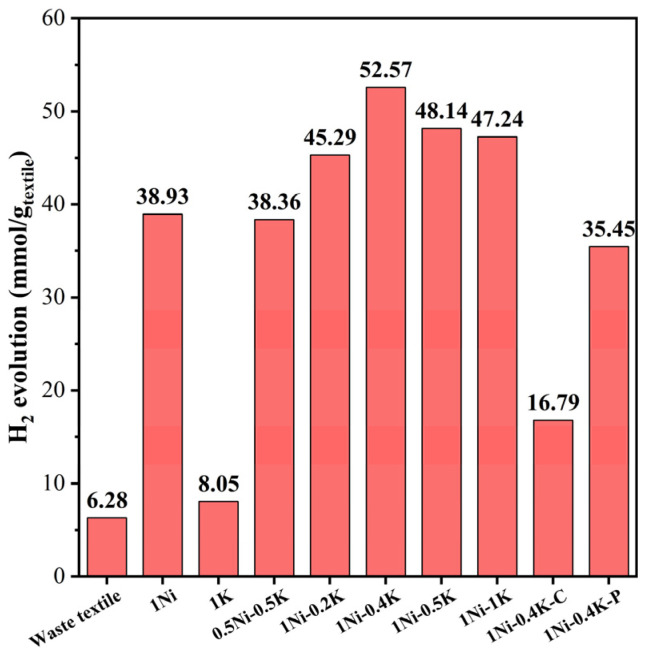
Effect of metal salt species, impregnation, and pyrolysis method on the catalysis yield of gas phase products and H_2_ evolution activities.

To further evaluate the process advantage of the impregnation method and microwave pyrolysis, taking the 1Ni-0.4K ratio as an example, the data of the physically mixed 1Ni-0.4K-P sample (microwave pyrolysis) and the 1Ni-0.4K-C sample (conventional pyrolysis) are considered (Figure 2). The results showed that conventional pyrolysis (1Ni-0.4K-C) gave a gas yield of only 20.35%. Physical mixing sample (1Ni-0.4K-P) gave a gas yield of 76.45%. The hydrogen production (Figure 3) of the impregnation method was 3.1 times that of conventional pyrolysis and 1.5 times that of physical mixing, and its H_2_ volume fraction (16%) was significantly higher than that of physical mixing (9%) and comparable to that of conventional pyrolysis (17%). This indicates that the impregnation method enables more uniform loading of metal salts inside the waste textile, and combined with the uniform heating characteristics of microwaves, significantly improves hydrogen selectivity and hydrogen production efficiency.

Figure 3 shows the amount of hydrogen produced per gram of waste textile after pyrolysis. The hydrogen production of pure waste textile was 6.28 mmol/g, which increased significantly to 38.93 mmol/g for the 1Ni sample, while the 1K sample produced only 8.05 mmol/g. Among all co-loaded samples, the hydrogen production was higher than that of samples with only Ni or only K. 1Ni-0.2K gave 45.29 mmol/g, 1Ni-0.4K reached the highest value of 52.57 mmol/g, 1Ni-0.5K gave 48.14 mmol/g, and 1Ni-1K gave 47.24 mmol/g. Compared with the 1Ni sample, under the same total metal loading, replacing 0.5 wt.% Ni with K resulted in little change in hydrogen yield. This indicates a synergistic effect between Ni and K when they are co-loaded. Compared with conventional pyrolysis (16.79 mmol/g) and physical mixing (35.45 mmol/g, with H_2_ only 9%), the 1Ni-0.4K increased hydrogen production by 3.1 times and 1.5 times, respectively, and also showed higher H_2_ selectivity. In summary, the 1Ni-0.4K ratio exhibited the best performance in terms of gas yield (67.47%), hydrogen production (52.57 mmol/g), and H_2_ selectivity (16%), while also having the lowest liquid yield among all samples (13%). 

**Figure 4:**
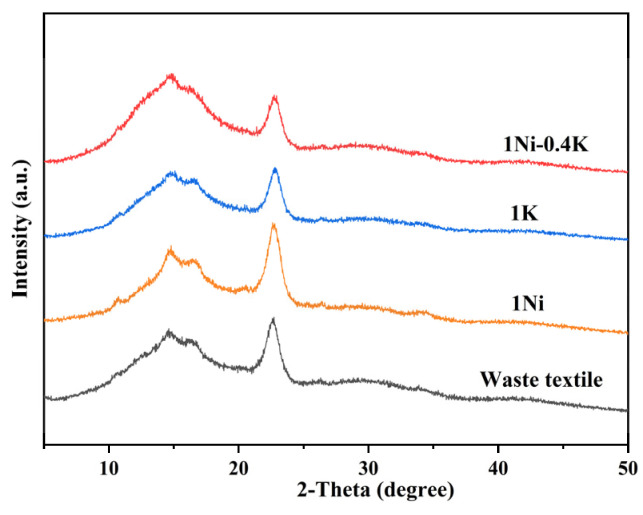
XRD patterns of waste textile, 1Ni, 1K, and 1Ni-0.4K samples.

To clarify the phase composition of metals loaded on waste textiles before and after microwave pyrolysis and to investigate the effect of alkali metal K on the Ni-containing textile, X-ray diffraction (XRD) was used to analyze the solid products. The unreacted samples (Figure 4) exhibited broad diffraction peaks at 15° and 22°, attributed to the structure of cellulose [24]. No additional diffraction peaks beyond these broad peaks were detected for the pure waste textile. The diffraction patterns of the 1Ni, 1K, and 1Ni-0.4K samples nearly overlapped with that of the pure waste textile, with no distinct crystalline diffraction peaks of metal salts observed. This indicates that under the loading levels and impregnation conditions used in this study, both nickel acetate and potassium acetate existed on the waste textile surface as highly dispersed amorphous or nanocrystalline phases, without forming X-ray-detectable crystalline phases. Moreover, the loading of metal salts did not alter the phase structure of the textile substrate. 

**Figure 5:**
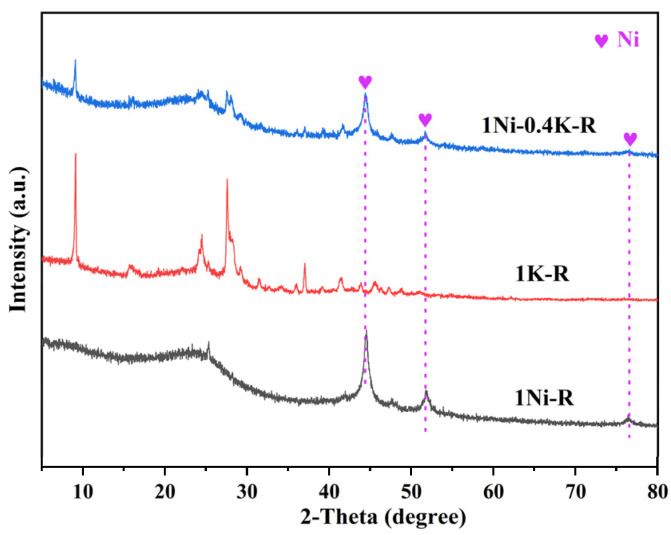
XRD patterns of 1Ni-R, 1K-R, and 1Ni-0.4K-R after microwave pyrolysis.

Compared with the pre-pyrolysis samples, the diffraction patterns of the post-pyrolysis samples (Figure 5) showed significant changes. All post-pyrolysis samples exhibited a broad diffraction peak in the range of 22–28°, which is attributed to the structure carbon [10]. 1K-R sample showed the diffraction peaks at 8.98, 24.5°, and 27.5°, assignable to potassium-based crystalline phases, indicating that potassium acetate was transformed into potassium species after microwave pyrolysis (such as K_2_CO_3_ or KHCO_3_ complexes) [9]. The 1Ni-R sample showed distinct diffraction peaks at 44.5°, 51.8°, and 76.4°, consistent with the standard PDF card 04-0850 for metallic Ni^0^, corresponding to the (111), (200), and (220) crystal planes, respectively, indicating that nickel existed in the zero valent metallic state [9,10,25]. The Ni diffraction peak positions of the 1Ni-0.4K-R sample were the same as those of 1Ni-R, and same potassium-containing crystalline phases were detected. Compared with 1Ni-R, the intensities of the Ni diffraction peak in 1Ni-0.4K-R decreased significantly (by 25–30%), and the full width at half maximum (FWHM) increased. Ni crystallite size was calculated using the Scherrer formula from the (111) peak, giving a result of 28 nm for 1Ni-R and 21 nm for 1Ni-0.4K-R. This result indicates that the introduction of K effectively inhibited the sintering and agglomeration of Ni particles during microwave pyrolysis through a steric hindrance effect, improving the dispersion of Ni active species.

**Figure 6:**
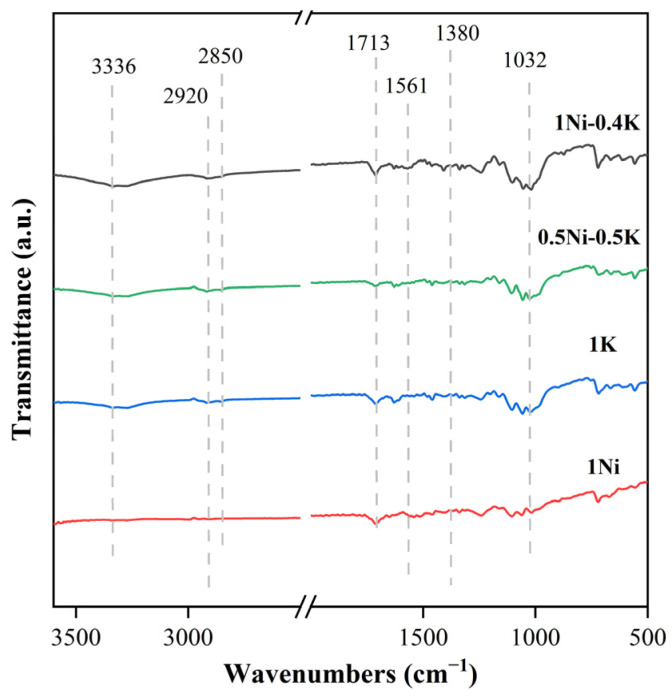
FTIR spectra of 1Ni, 1K, 0.5Ni-0.5K, and 1Ni-0.4K samples.

To investigate the evolution of functional groups in waste textiles loaded with metal salts during microwave pyrolysis, Fourier transform infrared spectroscopy (FTIR) was performed on samples before and after pyrolysis. Figure 6 shows the FTIR spectra of the samples before pyrolysis. All pre-pyrolysis samples exhibited a broad and strong absorption band near 3336 cm^−1^ with low transmittance, which is attributed to the stretching vibration of hydroxyl groups (–OH), mainly from hydrogen bonded water or free hydroxyl groups in cellulose and hemicellulose of the fabric fibers [24,26]. Two small peaks at 2920 cm^−1^ and 2850 cm^−1^ correspond to the symmetric and asymmetric stretching vibrations of C–H bonds in methyl (–CH_3_) and methylene (–CH_2_–) groups, indicating the presence of aliphatic chains in the fabric [27,28]. The shoulder peak near 1713 cm^−1^ can be assigned to the C=O stretching vibration of acetyl or ester groups in hemicellulose [28,29]. The bands at 1380~1630 cm^−1^ were related to C–O in acetate ions (CH_3_COO^–^) or the C–H bend vibration of methyl groups, confirming that the metal acetates were successfully loaded onto the waste textile [10,29]. The strong absorption band near 1032 cm^−1^ corresponds to the stretching vibration of C–O–C glycosidic bonds or C–OH in cellulose [30]. Comparing the samples impregnated with different metal salts, all Ni-containing samples (including 1Ni, 0.5Ni-0.5K, and the 1Ni-xK series) showed generally higher transmittance at 1032 cm^−1^ and 2920 cm^−1^. It suggests that Ni may have a coordination effect with the hydroxyl groups of cellulose, leading to the breakage of some hydrogen bonds.

**Figure 7:**
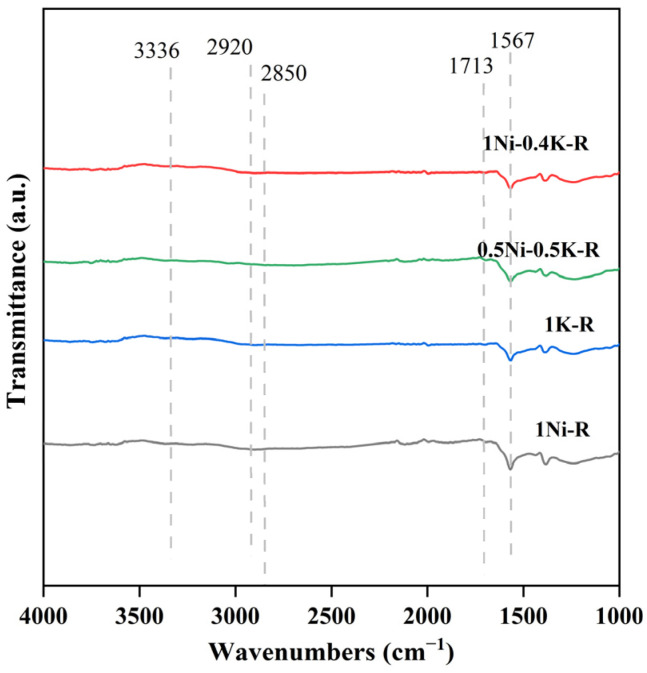
FTIR spectra of 1Ni-R, 1K-R, 0.5Ni-0.5K-R, and 1Ni-0.4K-R samples.

Figure 7 shows the FTIR spectra of the residues after microwave pyrolysis. Compared with the pre-pyrolysis samples, the characteristic peaks of C–H (2920 cm^−1^ and 2850 cm^−1^) and C=O (1713 cm^−1^) in all post-pyrolysis samples were significantly weakened or even disappeared, indicating that the aliphatic chains and oxygen-containing functional groups in the fabric underwent deep cracking during microwave pyrolysis. Meanwhile, the intensity of the O–H peak near 3336 cm^−1^ decreased substantially, confirming the destruction of the cellulose structure and the breakage of glycosidic bonds. All post-pyrolysis samples displayed a peak near 1567 cm^−1^, with significantly stronger intensity compared with the pre-pyrolysis samples. Before pyrolysis, the peak in this region mainly came from the asymmetric stretching vibration of acetate ions. Since nickel acetate and potassium acetate were largely decomposed at 500 °C, the original acetate characteristic peaks should have disappeared. Therefore, this peak is attributed to the C=C stretching vibration of aromatic structures formed by carbon skeleton reconstruction during pyrolysis. This indicates that the catalytic effect of nickel promoted the conversion of the carbon skeleton in the fabric toward aromatic structures, resulting in a more stable biochar structure. Lin et al. also found in their study on hydrothermal carbonization of waste textiles that a large number of aliphatic compounds decomposed while aromatic carbon increased significantly, confirming the general occurrence of carbon skeleton conversion toward aromatic structures during thermochemical conversion [26]. The potassium-containing sample (1K-R) still retained some C–H peaks after pyrolysis, indicating that the addition of potassium alone had a limited effect on the cracking of aliphatic chains. For the 0.5Ni-0.5K-R sample, the Ni loading was only half that of 1Ni, but the residual intensities of the C–H (2920 cm^−1^) peak after pyrolysis did not increase proportionally. They were similar to those of 1Ni-R, and the intensity of the aromatic C=C peak (1567 cm^−1^) was also comparable. This indicates that the introduction of potassium partially compensated for the loss of catalytic activity due to the reduced Ni content, therefor there is a synergistic catalytic effect between K and Ni. Potassium may promote the dispersion of Ni and assist in enhancing the ability of Ni to break C–H and C–O bonds. Therefore, the addition of potassium has a promoting effect on the pyrolysis of Ni-containing waste textiles, improving the degree of cracking, increasing gas yield, and enhancing hydrogen selectivity. FTIR analysis confirms the efficient catalytic role of nickel acetate during microwave pyrolysis, while the synergistic introduction of potassium acetate further optimizes the pyrolysis performance.

**Figure 8:**
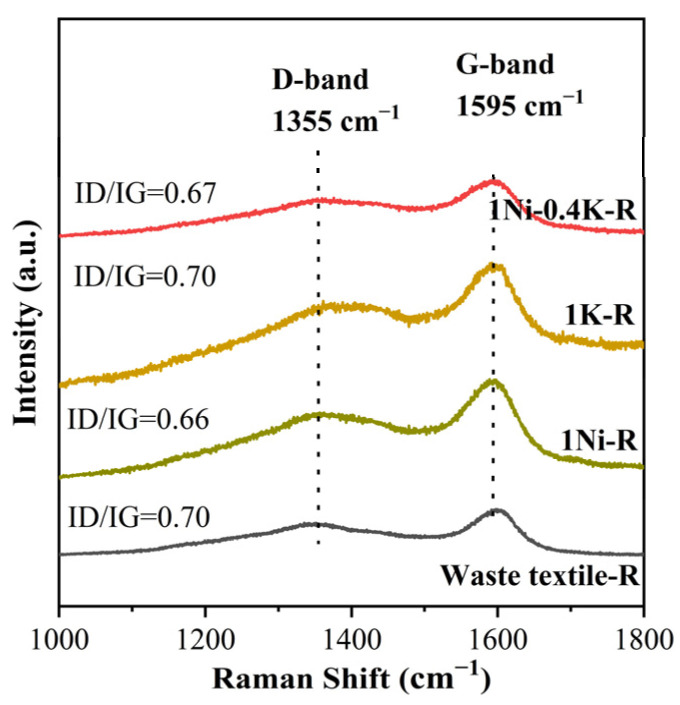
Raman spectrum of Waste textile-R, 1Ni-R, 1K-R, and 1Ni-0.4K-R samples.

The Raman spectra of the obtained materials after the pyrolysis test were obtained and the results are shown in Figure 8. The Raman spectra of the solid residues exhibited typical D and G bands at around 1355 and 1595 cm^−1^, respectively, confirming the formation of carbonaceous materials [9]. The decreased ID/IG ratio after Ni addition indicates an enhanced graphitization degree and a more ordered carbon structure. This suggests that Ni promotes carbon framework reconstruction and aromatization while facilitating C–H bond cleavage and dehydrogenation reactions, in agreement with the FTIR results.

**Figure 9:**
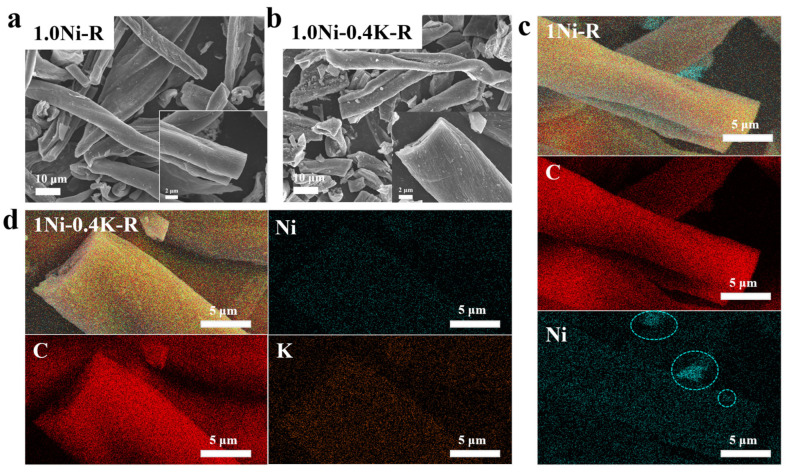
SEM images of 1Ni-R (**a**) and 1Ni-0.4K-R (**b**). SEM-Mapping of 1Ni-R (**c**) and 1Ni-0.4K-R (**d**). The dotted circles highlight the regions of Ni particle agglomeration.

The SEM images (Figure 9a,b) revealed that the surface of 1Ni-R was relatively smooth after microwave pyrolysis, while 1Ni-0.4K-R contained abundant small fragments and irregular particles. Combined with the Raman analysis, these results indicate that Ni promotes the formation of highly ordered carbon structures, whereas the introduction of K suppresses carbon graphitization to a certain extent. SEM-mapping (Figure 9c,d) further demonstrated the presence of noticeable Ni agglomerates in 1Ni-R, whereas Ni and K were homogeneously distributed throughout 1Ni-0.4K-R. The enhanced dispersion of Ni induced by K may increase the number of accessible catalytic active sites and inhibit Ni aggregation during the reaction process.

## 3. Experiment

### 3.1. Materials and Metal-Loaded Waste Textile Preparation

**Figure 10:**
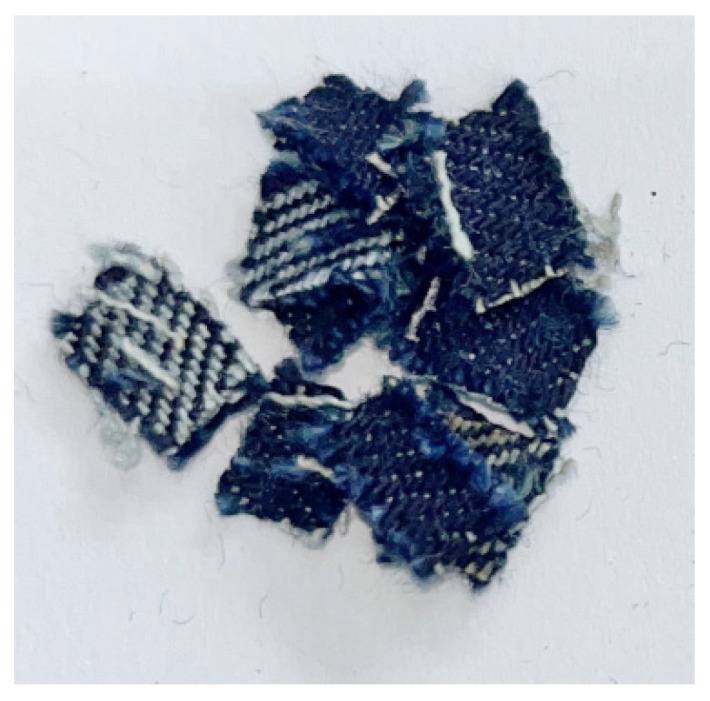
Waste textile used in the experiment.

Dark blue textile waste was obtained from the Haizhu Fabric Market in Guangzhou, Guangdong, China (Figure 10). The waste textiles were cut into 4 × 4 mm squares and dried in an oven at 110 °C for 12 h prior to testing. The metal salt (acetate) catalysts used in the experiment, NiC_4_H_6_O_4_·4H_2_O and CH_3_COOK, were purchased from Shanghai Aladdin Reagent Co., Ltd., Shanghai, China (analytical grade). The catalyst was loaded by impregnation [10,31]. The procedure was as follows: The waste textile was cut into small pieces to obtain fresh waste textile samples. A total of 8.0 g waste textile sample and a specified amount of acetate (nickel acetate, potassium acetate, or their mixture) were added into separate beakers. Then, 22 g deionized water was added to dissolve the acetate, after which the textile waste was immersed for a set period. After impregnation, the samples were dried in an oven at 110 °C for 16 h and stored in a desiccator for later use. Samples with different Ni/K ratios were thus prepared. The metal loadings on the textile waste were determined using an atomic absorption spectrophotometer (AA-7000, Shimadzu Corporation, Shimadzu, Japan). The results showed that no detectable Ni or K species were present in the untreated textile waste. After impregnation, the measured metal contents were in good agreement with the designed loading values, with deviations generally within ±5%. These results confirm that the impregnation method achieved effective deposition of metal species onto the textile substrate and that the actual metal contents closely matched the target loadings. Hence, the textile waste without metal salt addition was labeled as Waste textile. Samples containing 1 wt.% Ni or 1 wt.% K were labeled as 1Ni and 1K, respectively. Samples with a fixed Ni loading of 1 wt.% and varying K addition of x wt.% (x = 0.2, 0.4, 0.5, 1) were denoted as 1Ni-xK. The sample co-loaded with 0.5 wt.% Ni and 0.5 wt.% K was denoted as 0.5Ni-0.5K. Post-pyrolysis samples were denoted by adding the letter R after the corresponding label, e.g., Waste textile-R. The physically mixed sample (the loading method was changed to physical mixing) was labeled as 1Ni-0.4K-P, and the conventional pyrolysis sample was labeled as 1Ni-0.4K-C. 

### 3.2. Microwave Pyrolysis Tests

**Figure 11:**
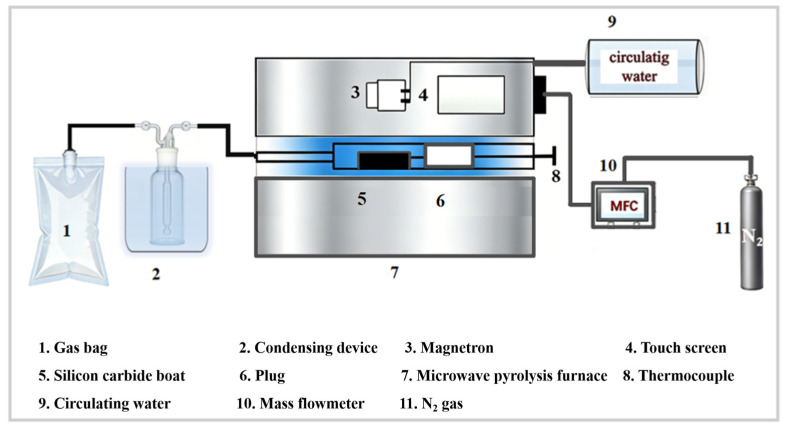
Schematic diagram of the microwave pyrolysis system.

The microwave pyrolysis tests of the samples used in this experiment were carried out in a CY-PY1100C-S microwave pyrolysis furnace (Changyi Microwave Technology Co., Ltd., Changsha, China). The equipment operates at a frequency of 2450 ± 50 MHz and an output power of 1.4 kW, providing a controllable nitrogen atmosphere and a stable high-temperature reaction environment. Its system structure is shown in Figure 11. Before the experiment, 1.0 g sample was placed into a silicon carbide boat, and the boat was then placed into the microwave pyrolysis furnace. Subsequently, high-purity nitrogen (99.9% purity) was introduced into the quartz reactor at a flow rate of 100 mL/min for 15 min to fully expel the air from the reactor and create an inert atmosphere. Setting the appropriate temperature conditions (maintaining a nitrogen flow rate of 40 mL/min during the reaction), the temperature was raised to 500 °C and held at this temperature for 5 min. For comparison, conventional pyrolysis was performed at 500 °C for 5 min. The microwave power was automatically adjusted based on real time temperature to achieve precise temperature control. The gaseous products generated by pyrolysis were collected in a polytetrafluoroethylene gas bag after condensation, the pyrolysis oil was collected in a receiving bottle and weighed after cooling to room temperature, and the solid residue (pyrolysis solid) was taken out and weighed after the reactor had cooled naturally. The contents of the gas, liquid, and solid three-phase products were calculated. Gas components were detected by a gas chromatograph (GC9790Plus, Zhejiang Fuli Instruments Co., Ltd., Zhejiang, China). To evaluate the catalytic performance, the solid, liquid, and gas yield are calculated as follows:

(1) $$Solid\;yield\;(\%)=\frac{m_{solid}}{m_{obtained\;materials}}\times 100\%$$

(2) $$Liquid\;yield\;(\%)=\frac{m_{liquid}}{m_{obtained\;materials}}\times 100\%$$

(3) $$Gas\;yield\;(\%)=\frac{m_{obtained\;materials}-m_{solid}{-m}_{liquid}}{m_{obtained\;materials}}\times 100\%$$

where $\;m_{obtained\;materials}$ (g) is the mass of raw or metal-containing waste textiles, $m_{solid}$ (g) is the mass of residue solid after microwave pyrolysis test, $m_{liquid}$ (g) is the mass of liquid collected during the microwave pyrolysis test.

The pyrolysis performance is characterized by the composition and yield of the gaseous products. The volume fraction *Yi* (%) of a component refers to the volume fraction of that component in the syngas, and this study mainly focuses on the hydrogen volume fraction. Its calculation formula is as follows:

(4) $$Y_{i}=\frac{x_{i}}{x_{H_{2}}+x_{CO}+x_{{CO}_{2}}+x_{{CH}_{4}}+x_{C_{2}H_{m}}}$$

where *x_*i*_* (vol.%) is the percentage of gas volume detected by GC.

### 3.3. Characterization

The crystal structure of the solid products was analyzed by X-ray diffraction (XRD, D8 Advance, Bruker Corporation, Karlsruhe, Germany). The chemical bonds and functional groups were characterized using a Fourier transform infrared (FTIR) spectrometer (IRTracer-100, Shimadzu Corporation, Shimadzu, Japan). Before FTIR analysis, the sample was pretreated in an oven at 110 °C for 12 h to remove residual moisture. The metal loadings on the samples were determined using an atomic absorption spectrophotometer (AA-7000, Shimadzu Corporation, Shimadzu, Japan). Prior to analysis, a known amount of sample was immersed in a prepared acid solution and subjected to ultrasonic treatment for a certain period to ensure complete dissolution of the metal species deposited on the textile substrate. The resulting solution was subsequently filtered and diluted to an appropriate concentration before measurement. The microstructure and element mapping of catalysts were characterized by scanning electron microscopy (SEM, Sigma 500/VP, Carl Zeiss AG, Oberkochen, Germany). The carbon structure of the solid residues obtained after microwave pyrolysis was characterized using a Raman spectrometer (LabRAM HR Evolution, HORIBA Scientific, Kyoto, Japan).

## 4. Conclusions

In this study, the effect of K introduction on the catalytic pyrolysis performance of Ni-containing waste textile was investigated using microwave-assisted catalytic pyrolysis. The results showed that neither 1Ni nor 1K alone increased the gas yield compared with pure textile, whereas the co-loading of Ni and K significantly enhanced the gas yield, with the 1Ni-0.4K sample achieving the highest gas yield. The K loading amount played a key role in regulating hydrogen production, and the highest hydrogen yield of 52.57 mmol/g was achieved at a K loading of 0.4 wt.%. XRD and SEM-mapping analyses confirmed that K addition effectively inhibited Ni agglomeration and improved Ni dispersion. Raman and FTIR results revealed a synergistic interaction between Ni and K, whereby K compensated for the effect of reduced Ni loading. Moreover, Ni promoted the formation of a more ordered carbon structure, facilitating hydrogen-generating reactions during microwave pyrolysis. These findings provide new insights into the design of alkali-promoted Ni catalysts for efficient hydrogen production from textile waste.

## Data Availability

The original contributions presented in this study are included in the article. Further inquiries can be directed to the corresponding authors.
